# “Staying for the children”: The role of natal relatives in supporting women experiencing intimate partner violence during pregnancy in northern Tanzania – A qualitative study

**DOI:** 10.1371/journal.pone.0198098

**Published:** 2018-06-01

**Authors:** Geofrey Nimrod Sigalla, Declare Mushi, Tine Gammeltoft

**Affiliations:** 1 Institute of Public Health, Kilimanjaro Christian Medical University College, Moshi, Tanzania; 2 Department of Health, Evangelical Lutheran Church in Tanzania, Arusha, Tanzania; 3 Department of Anthropology, University of Copenhagen, Copenhagen, Denmark; University of North Carolina at Chapel Hill, UNITED STATES

## Abstract

**Introduction:**

Intimate partner violence (IPV) is a global health and human rights problem. In Tanzania, national studies have shown that half of all women experience partner violence in their lifetime, 38% reported being abused during a period of 12 months and 30% during pregnancy. Despite the benefits of social support to women victims of violence during pregnancy, a majority of women hesitate to seek help and, if they do, they mainly turn to their natal relatives for support. However, this process of help-seeking and the type of support received is not well documented and needs to be explored with a view to future interventions. This article investigates women’s own perspectives on the support they receive from natal relatives when experiencing IPV during pregnancy.

**Materials and methods:**

Eighteen participants who experienced physical IPV during pregnancy were purposively selected from a cohort of 1,116 pregnant women enrolled in a project that aimed at assessing the impact of intimate partner violence on reproductive health. In-depth interviews were used to explore the social support received from the natal family among women who experienced partner violence during pregnancy. All interviews were audio recorded, transcribed, coded and analyzed.

**Results:**

Women who experienced severe IPV during pregnancy were more likely to seek help from natal relatives. Severe violence was defined by the women as acts that occurred frequently and/or resulted in injury. The women’s natal relatives were willing to provide the support; however, they strongly encouraged women to maintain their marriage so that they could continue caring for their children jointly with their partners. Emotional support was the commonest form of support and included showing love and empathy and praying. Information provided to victims aimed mainly at advising them to maintain their marriage. Practical support included direct financial support and building their economic base to reduce dependency on their partners. When the couple was on the verge of separation, mediation was provided to save the marriage.

**Conclusion:**

Women who experienced partner violence preferred to seek help from their natal relatives. The support provided by natal relatives was beneficial; however, maintaining the marriage for the care of children and family was given the highest priority, over separation. As a consequence, many women continued to live with violence. Stakeholders supporting victims of violence need to understand the priorities of victims of violence and structure intervention to address their needs.

## Introduction

Intimate partner violence (IPV) is a global health and human rights problem that include physical, sexual and psychological harm [1]. IPV disproportionately affects women relative to men [2]. Globally, one in three women report having experienced IPV in their lifetime, with prevalence higher in African countries [3]. In Tanzania, recent studies estimate the lifetime prevalence of IPV to range from 39 to 65% [4–6]. A population-based survey done in 2015 showed that half of ever-partnered women aged 15–49 years had experienced physical or sexual violence in their lifetime, and 38% reported being abused during a period of 12 months prior to the survey [5]. The Tanzania Demographic and Health Survey 2015 further showed that the prevalence of physical and sexual violence was 39% and 14% respectively.

IPV is also a problem during pregnancy in Africa where a review of African studies on violence showed that up to 57% of women had experienced IPV during pregnancy [7]. In Tanzania, the prevalence of IPV during pregnancy is high, ranging from 8.1% to 30% [5,6]. In Moshi, Kilimanjaro, from where the sample of this population was drawn, 30.2% of women experienced IPV during pregnancy, with 22.8%, 6.3% and 15.3% reporting emotional, physical and sexual IPV respectively [8]. IPV is associated with serious health consequences to the victims, such as symptoms of anxiety, posttraumatic stress disorders and depression [6,9,10], elevated risks of HIV infection and progression to AIDS [11], and adverse outcomes of pregnancy that include stillbirth, preterm and/or low birth weight delivery [12–14]. In Tanzania, physical violence is an offence and is covered by criminal laws. There is no separate law that addresses IPV against women and the Sexual Offences Special Provision Act (SOSPA) does not include a provision for marital rape [15].

Social support is known to be beneficial to women who have experienced partner violence [16–20]. First, social support is associated with decreased risk of experiencing IPV during pregnancy [8]. Social support reduces the levels of stress and distress caused by violence [16]. It also mitigates the negative mental health impact of stress that is associated with violence, contributing to women’s overall well-being [17]. Social support has also been reported to have positive health benefits among women who experience IPV during pregnancy [18,19]. A study conducted in Iran showed that women who experienced IPV but had adequate social support during pregnancy delivered babies with higher birth weights when compared to those who had low levels of social support [18]. Conversely, lack of social support has been reported to be associated with higher risk of pregnancy complications such as miscarriage, pre-eclampsia and preterm births [19]. In general, studies show that social support improves the quality of life of women exposed to partner violence [20].

Research has shown that the majority of victims of IPV seek support from informal support network members (e.g. family, friends, neighbors, classmates, work-mates) [21] and support from the natal family is the most utilized [22]. A review of studies on social support has shown that natal family support to victims of IPV range from emotional support, advice and information to practical/tangible support. However, many other authors have observed that the type of support received by the victims of violence when seeking help is not well understood and have recommended further exploration [21,23,24]. Basing on theoretical framework for help-seeking among victims of intimate partner violence, the Cognitive Theory puts forward three stages which are important for someone experiencing violence to seek help [23]. The victim defines violence as a problem followed by deciding that help should be sought and finally, selects source of support. Qualitative interviews are appropriate in exploring this subject considering the sensitivity around gender based violence [25], and in establishing the context where violence occurred, victim’s decision-making process to seeking help from natal relatives and the actual support received. Qualitative interviews will have the advantage of building trust with interviewees, enabling the participants to share their personal experiences and describe their perceptions and decisions in their own words.

In Moshi town, Tanzania, where this study was conducted, the declining economic activities in the rural areas, especially the once robust economy from coffee, has increased the urban migration to Moshi and creates further constraints for the residents of the municipality. Moshi Municipality is known for its tourism industry due to being in the vicinity of the Kilimanjaro Mountain and Ngorongoro and Serengeti national parks. Most women who have been rearing cattle and growing bananas in rural areas are now trying to engage in small businesses or stay as housewives without income. The common household strategy is for the husband to live with the female partner either alone or close to/with his parents. Unmarried young mothers, together with their children, continue to live with and depend on their natal parents. The Chaga men are known in most Tanzanian towns for their business skills. Traditionally, men would leave their wives and children in the village to rear cattle and coffee while they went to establish businesses far away. However, this pattern is now changing because most women also live in town and are actively engaged in small businesses or employment [26]. In all of the living arrangements described above, natal relatives continue to be part of these women’s lives, irrespective of where couples live, proximity to natal relatives or male partner’s economic status. Women do increase their reliance on natal relatives, from what is common for women in this cultural setting, to seek the advice of their natal mothers and sisters during pregnancy and childbearing. Therefore, many women view their natal family as an integral part of their lives and, most often, members of the natal family continue to play important roles in women’s daily lives regardless of their marital status [27]. However, it is not clear what support the natal family provides to women who are victims of partner violence. This article therefore aims to explore the support provided by natal relatives to women experiencing IPV during pregnancy in Moshi, Tanzania.

## Materials and methods

### Ethics statement

Ethical approval of the study was obtained from the Kilimanjaro Christian Medical University College Research and Ethical Review Committee (CRERC), certification number 592, January 17, 2014 extended yearly. Permission was sought from the Executive Director of Moshi Municipality to carry out research in the district. We followed the WHO ethical and safety recommendations when doing research on violence against women. All participants were informed about the study and approved participation through signed consent. Support services were available for women who needed further help and were provided after their approval to be referred. Support services included those related to legal, health, child support and police issues.

### Study design and setting

In-depth interviews were used to explore the social support received from the natal family among victims of partner violence during pregnancy. Participants in this study were purposively selected among those who participated in a larger cohort study that assessed the impact of intimate partner violence on women’s reproductive health. The cohort study included 1,116 pregnant women and formed part of PAVE project, a multi-country study that aimed at generating insights into the prevalence, forms, and consequences of intimate partner violence. Since the focus of the larger research project was on women’s exposure to partner violence during pregnancy, the present study also focused on pregnant women.

In Tanzania, the study was conducted in Moshi municipality, which is one of the seven districts in Kilimanjaro region, in the northern part of the country. It has an estimated population of 206,728 people, with annual population growth of 2.8%, which is mainly attributed to rural–urban migration. Among members of the Chaga and Pare tribes, where this study is focused, kinship is patrilineal and patrilocal and children are considered to be “owned” by the male partner [28]. Polygamy is not commonly practiced except by the few Muslims in the area. At marriage, a dowry is paid by the family of the male partner to the woman’s natal family to seek approval of the union. After the marriage ceremonies, the woman usually moves to live with her partner together with or near his family. The partner’s family members are then responsible for keeping an eye on the daily affairs of the new couple, including settling marital disputes [29].

### Study participants and data collection

Eighteen participants were purposively selected among the 1,116 pregnant women enrolled in the cohort study. The study was conducted from March 2014 to May 2015. Details of this cohort study is described elsewhere [8] but briefly, pregnant women were enrolled from two antenatal care clinics before the 24^th^ week of gestation, followed at the 34^th^ week of pregnancy and within 48 hours of delivery. Women who participated in the cohort were aged 18 years or above, with a singleton pregnancy, who delivered within Moshi Municipality and who were willing to be followed for the entire period of the study.

Enrolled to the present qualitative study were women who reported having experienced physical IPV during pregnancy. Participants with other forms of partner violence (sexual and/or emotional) were included in the study provided that they had also experienced physical violence from their partner during pregnancy. Building on the results from the cohort study, the present qualitative study aimed to further explore the life situations of women who had experienced physical violence during pregnancy. Experiencing physical violence during pregnancy was associated with unwanted birth outcomes of preterm birth and low birth weight [13]. Victims of physical violence during pregnancy were also more likely to disclose their experience to natal relatives than those with other forms of partner violence [22]. This sample was therefore selected to understand their experiences with partner violence, their decision-making process for disclosure to natal relatives and support received. The sample of women included women who were 18 to 39 years old, with varying education level (primary education/secondary education), occupation status (employed, business or housewife), marital status (married or never married) and number of children a woman had (from no child to seven children). Data were collected through in-depth interviews conducted by two researchers, one male and one female. The male researcher is a medical doctor, with ten years’ experience in providing reproductive health services to clients and is the first author of this article. Prior to conducting the field research, the research team had carefully considered whether a male researcher would be able to establish the necessary rapport with women living with partner violence. During data collection, comparisons between the interviews conducted by the male and female researcher soon indicated that respondents offered their stories in as much detail and depth to the male as to the female interviewer. It was therefore concluded that with respect to the validity of the interviews, the interviewer’s training, personality and capacity to listen in an empathetic way seemed more important than his/her gender. Similar conclusions regarding use of male interviewers have been documented in other gender based violence studies [25,30]. Further, prior to conducting the qualitative interviews, both researchers had received targeted training in the conduct of research on sensitive topics. A semi-structured interview guide with open-ended questions was used during the interview. The interview guide included questions on the forms of partner violence experienced by the participant, what compelled them to seek help, and details of the social support they received from natal relatives and others. After every interview, the collected information was reviewed and discussed by research team so that any new issue raised could guide the next interview.

Most of the interviews were done in a separate room at the clinic to ensure privacy and confidentiality. For women who preferred to be interviewed at home, it was made sure that no one other than the participant and children under two years of age were present during the interview. All interviews were audio recorded with participant approval. The interviews were conducted in *Swahili* language, the language spoken by all participants. Baseline interviews lasted for a period of one to two hours and aimed to explore the experience of violence during pregnancy, help-seeking and support received from natal relatives. After interviewing the fifteenth participant, we felt that saturation was reached, a situation where additional information would not result into a new insight. To confirm that, three additional participants were interviewed, and review of the interviews resulted in generating no new information. Follow-up interviews were conducted with fourteen selected participants about two to three months after their first interview and lasted for about half an hour. These participants had reported experiencing repeated episodes of violence and therefore the follow up interview assessed their progress on the status of the violence they had reported, child care and support.

### Measures

In the cohort study, the assessment of the experience of IPV was done using the WHO questionnaire that had been used previously in Tanzania [31,32]. To assess physical violence, the women were asked if, during the index pregnancy, their partner had slapped, pushed, hit, kicked, choked or threatened to use or actually used any object that could hurt them. Emotional violence was defined as being insulted, humiliated, intimidated or threatened by the partner while sexual violence included being physically forced to have sexual intercourse, having sexual intercourse without consent or being forced to do a humiliating or degrading sexual act.

In this study social support was defined as any form of assistance that female victims of partner violence felt they received from other people; this could be from a trusted friend or supportive social networks such as their natal relatives or their partner’s family. Social support included emotional support, practical support, information and/or mediation. Socioeconomic characteristics were also assessed in the present study. Socioeconomic characteristics included age of participant, education level (not attended school/primary education/secondary education and above), occupation status (employed, business or housewife) and marital status (married or never married) and number of children.

### Analytical procedures

Field notes were written and expanded within twenty-four hours after the interview. All audio-recorded interviews in Swahili were transcribed verbatim by an experienced transcriber followed by English translation. The two authors (GNS and DM) are residents of Tanzania, speak Swahili and English fluently and frequently cross-checked the verbatim transcription and translation. After reading the transcripts, the two authors (GNS and DM) did preliminary open coding of text to identify common themes that emerged from the transcriptions. Examples of codes that emerged were help-seeking, emotional support, practical support, and information for support, reconciliation, support networks and child support. Authors agreed on the three themes for analysis; disclosure for support, social support and social support from natal relatives. This was followed by further coding of all transcripts manually (GNS). During the whole process of analysis, there was a constant checking of the text, codes and themes while comparing to the research questions for relevance. Outcomes of interest that were analyzed included help-seeking, circumstances that surrounded the women’s decision to seek help and the support received from their natal relatives and other people. In terms of theory, a Grounded theory approach was used: through close and systematic attention to the core themes brought up by the women during interviews, the authors developed the interpretations and insights that are presented in this article.

## Results

### Participants

A total of eighteen participants were included in this study. The participants’ ages ranged from 18 to 39 years and the majority (n = 10) were aged between 26 and 35 years (Table 1). Most participants (n = 13) had completed the primary level of education. Regarding occupation, two women were employed by the government as teachers and two others were employed by private companies as a cleaner and as a parking fee collector; seven women engaged in small businesses in a nearby market—selling secondhand clothes, cereals or seasonal fruits; one woman was a farmer and the remaining six were housewives. Nearly all participants (n = 16) migrated into town from their natal village. At the time of the interview, nearly three-quarters of the participants (n = 13) were married. Five participants were living with the family of their husband, while 13 were living in a nuclear family. While five participants were pregnant for the first time, seven already had one child and six had more than two children.

**Table 1:** Characteristics of participants (n = 18).

Characteristic	Number, n (%)
Age in years	
18–25	6 (33.3)
26–35	10 (55.6)
36–39	2 (11.1)
Age at first pregnancy in years	
Below 18	4 (22.2)
18 and above	14 (77.8)
Level of education status	
Primary school education	13 (72.2)
Secondary school education	5 (27.8)
Occupation	
Employed	4 (22.2)
Business	8 (44.4)
Housewife	6 (33.4)
Marital status	
Married and living with a partner	13 (72.2)
Never married but living with a partner	5 (27.8)
Where did they grow up?	
This commune	2 (11.1)
Another place in the village	16 (88.9)
Living with family of partner	
Yes	5 (27.8)
No	13 (72.2)
Number of living children	
0	5 (27.8)
1	7 (38.9)
2 or more	6 (33.3)
Physical violence during pregnancy	
a) Slapped or thrown something at that could hurt	11 (61.1)
b) Pushed or shoved or pulled your hair	8 (44.4)
c) Hit with his fist or with something else that could hurt	4 (22.2)
d) Kicked, dragged or beaten up	3 (16.7)
e) Choked or burnt on purpose	1 (5.6)
f) Threatened to use or actually used a weapon	3 (16.7)
Disclosed to the natal family the experience of violence	
Yes	10 (55.6)
No	8 (44.4)

### Experiences of partner violence during pregnancy

All eighteen participants experienced physical violence during pregnancy, and the majority (n = 15) had experienced repeated episodes of violence with frequencies ranging from two to seven times. Fourteen women reported to have experienced emotional violence from their partners and twelve women experienced sexual violence.

### Whether or not to seek support

A total of thirteen participants did seek help to end violence: seven had disclosed only to their natal relatives, two reported to the natal relative and a friend, one disclosed to a natal relative and a pastor, two reported to the partner’s family only and one shared her troubles only with a friend. In other words, ten of the women victims of partner violence relied on their natal relatives for support. Of these ten women, six resided with the partner’s family. The support provided by natal relatives was reported to be important to all women regardless of the living arrangements. Since the natal relatives were the most preferred support group by women who experienced violence, further exploration was made to understand the participants’ decision-making process while seeking support. Women victims of partner violence narrated that they went through serious consideration as to whether they should seek support from the natal family or not. Their decision-making was influenced by three main factors: the severity of the violence, feelings of inferiority and/or doubts regarding the natal family’s ability to help.

#### Severity of violence

Thirteen women reported that a woman should be tolerant of minor forms of violence from her partner as they occur frequently in their relationship. Eight women used the famous saying in Tanzania that “even two glasses in the cupboard may fall over one another” to illustrate how common violence can be in their relationships. It is usually only when such violence becomes frequent or severe enough to cause injury that women will opt to seek help from the natal family. A 22-year-old woman with primary school education said,

*I tried to tolerate his actions until one day when he injured my finger… so severely that it was deformed*. *Then I decided to tell my mother*. *I called her and narrated the whole story from when it started to what was happening at that point*. (IDI 14)

#### Feelings of inferiority

Participants who did not fulfill certain family obligations felt inferior and, as a result, they were less likely to seek help from their natal relatives. One woman felt that she was too much of a burden to her natal relatives as she had not fulfilled her cultural responsibility of bearing children. This woman explained that bearing children is one of the important aspects of being a woman in Chaga culture. Especially when a woman delivers a male child, she is more respected and cared for than when she delivers a girl child. When she failed to have any living child within six years of marriage she, described herself as being very disappointed and shaken. Although her situation of violence did not result from not having children, she felt that she could not ask her already distressed natal family for support in relation to the violence she endured in her life.

*I don’t tell my mother about these frequent beatings*. *She already has enough pain that I didn’t have children*. *Then how can I dare to tell her these other challenges*? *My mother may faint*! *I don’t know about other tribes but in Chaga*, *having children is very special and a wish of everyone—and especially so for a baby boy*. [A 39-year-old woman with secondary school education] (IDI 15)

Another situation that made women feel inferior, and not deserving of respect from their natal family, was when they didn’t fulfill their cultural obligation of formalizing their relationship. It was reported by a majority of participants (n = 10) that behaviors which were culturally inappropriate in their area included getting pregnant before marriage, living with the husband before getting approval by the natal family or when the initial installment of dowry was not fully paid. Illustrating how such situations will negatively influence women’s decision to seek support from their natal relatives, a 27-year-old woman with primary education, described her situation:

*Imagine*, *I am pregnant before marriage*. *I am living with him before he has paid the dowry… this is not culturally appropriate*. *Therefore*, *I can’t tell my parents about the abuse*. *I am even afraid to go back home with this pregnancy*. *When my mother sees me with this pregnancy*, *she will for sure faint even before she listens to my issues of violence*! (IDI 18)

#### Doubts about the natal family’s capacity to help

Among those who did not seek help, some responded that the poor conduct of their natal family was a factor that hindered them from seeking help. One participant wondered if, when the parents themselves do not live up to the standards of a good relationship, telling them will make any difference.

*I can see how my parents live in their relationship*, *it is worse than my situation*. *A fight among them is a usual thing*. *For example*, *when my father gets money*, *he will not come home for three or four days*. *He becomes rude and no one should ask him anything until all the money is gone*. *That is when he comes back home and calms down*. *Now such a family*, *complaining to them about the beating from my husband will make no sense*. *They may see it as a very simple problem that I don’t deserve to complain about*. [27-year-old woman with primary education] (IDI 02)

### Social support provided by the natal family

Analysis of reports from participants who did receive support from the natal family indicates that nearly all of them were encouraged to stay in their relationship. However, educated parents and young sisters and brothers asked women to consider their situation and encouraged that they make a decision to leave. The type of support provided were emotional support, practical support, information and/or mediation as described in detail below.

#### Emotional support

This was the most common form of support received by most participants. All participants reported that their natal family was ready to continue showing them love and empathy despite their partners thinking that they are of no value. When love was not expressed by the partner, one participant explained that she was looking for an alternative place to be loved and the natal family was ready to love her.

*Remembering when I called my father and described the situation [of violence]*, *he told me to come home since he still loves me very much*. *He said that I should try to find a reason to make my husband allow me to go back home and stay there for some time…so that I get a break*, *relax and calm my stress*. *After that call*, *I felt very good that my parents still love me very much… that I am still of value to them and that they heartily welcome me home… although my husband thought that I am of no value to him*. [A 32-year-old woman with primary education, and a housewife] (IDI 12)

However, being welcomed home to the natal family was not typical, as three other women reported that they were not welcomed. They narrated that in the area, once married they are expected to live with their in-laws and not with the natal relatives.

Stress caused by violence from the partner significantly affects women’s lives in marriage and some reported having thought of leaving the relationship. They reflected back to the sense of belonging they had with their natal family before starting their relationship with the current partner. One woman reported that, at times of trouble, she was continuously encouraged by her natal family when they indicated to her that she was still their daughter. She felt that the intimacy, affection and warmth from her mother made her forget all of the challenges in her relationship with her partner.

Four participants said that when all hope was gone in their life, the only hope comes from God. Anyone who joins them to pray together for better relationships in their lives are seen as very helpful partners. Three participants reported that the natal family is a key partner in prayers and they pray for all family members. This is especially so if the parents are believers in Christianity. One participant even described how, when she requests her mother to pray for her, it implies that she is in trouble and her mother prays for her. The 23-year-old woman with primary education, described her hope in prayers,

*There is hope in prayers*, *I tell you*. *Satan is very strong in interfering with God’s plan between the two of you in your relationship*. *Loving parents should hold you in their prayers and my mother is very good in praying*. *Once I tell her to pray for me*, *she knows I am in trouble and she starts praying*. *I can tell you that prayers work and my husband comes home the next day changed*. *Then I believe that the prayers have helped*. (IDI 17)

#### Information/Advice

Three participants who did seek help from natal relatives reported that there was a difference in the advice provided to them by the natal parents as compared to that from other relatives of relatively similar age as the victim. Natal parents preferred that they stay longer in a relationship and advised them to tolerate further. However, other members of the natal family advised the victim to consider the situation and take appropriate action, which may include leaving the abusive relationship. When comparing the advice she received from her mother and that from her sister, a 28-year-old woman working in business narrated,

*My sister was advising me*. *She told me that*, *if my life in this relationship is difficult and I can’t tolerate it*, *I may opt to leave and go back home to restart afresh*. *She was very open and frank with me…this was different from what my mother was advising me*, *to stay in the relationship because that is what men are*. (IDI 03)

One participant reported having been advised by her sister to stand up for herself and tell her partner the truth, that she cannot tolerate the abuse. She provided information to her of other options available to seek support in order to stop the violence. She felt empowered and decided to confront her partner. This 31-year-old woman, employed in a private company, had the following to say:

*After my sister advised me to speak to him [the abusive partner]*, *I took the move to tell him the truth*. *I told him how abusive he was to me*. *I shared with him the blessings God has given in our lives*, *including children*. *However*, *I tabled two options for moving forward*: *that he either stop beating me or I leave the relationship to start my own life after taking legal action on him*. *He felt bad and promised to change*. *He started going to church*, *stopped taking alcohol and life started coming back to normal*. (IDI 04)

#### Practical support

Many participants expressed the belief that caring for children was the crucial role of women in their area. Safety and support of children was at the center of most women’s lives, including those who didn’t seek support from the natal family. Poor financial situation of their natal family makes it hard for them to support the woman plus her children in case she leaves the abusive partner. Women themselves were not better financially to support their children without external support. Three women reported that they were therefore forced to continue suffering in the relationship just for the sake of children, so that they stay supported by the partner. They explained that it was very expensive to take care of their children in terms of food, clothing and school fees when they are of school-going age. Although there are laws to govern the arrangements for child support, one woman explained how she was dissatisfied with their enforcement. On the other hand, she has received an offer from her natal family to support her children if she decides to leave the abusive relationship.

*My father once said*, *my daughter*, *you leave him [the partner] and come home*. *We will make sure that we take good care of these children including taking them to school*. *We will try our level best to find money for their support*. [A 31-year-old woman with primary level education] (IDI 09)

Most women (n = 10) reported that the experience of physical violence was accompanied with limitations in financial support that were aimed at causing more suffering and/or limiting their ability to leave the abusive relationship. However, in many cases, the natal family was there to provide money for expenses such as food and child care. However, all women who reported receiving financial support from the natal family stressed that the partner didn’t know about such support being provided.

*For example*, *after beating me*, *he would go to work without even leaving a cent while knowing that I have no work and have nothing to buy food for me and the children*. *I really depended on my sister who was ready to listen to my difficulties and give me money for food*. *He would then come home that evening*, *eat food and sleep*, *without even asking where I was getting money from*. *That is how life goes*. [28-year-old woman with primary education] (IDI 06)

Four women reported that they had been supported by their natal family with capital to start their business so that they were not economically dependent on the abusive partner. These women explained that the offer came after they disclosed the abuse to the natal family, especially when such a quarrel was on finance matters. Only one out of these four women revealed the offer to her partner, but only because she was given sewing machine and needed his approval. Three women tried to develop alternative explanations as to where such support was solicited. Alternative explanations included a loan or support from a friend. Two reported that they had to disguise the help by requesting a smaller amount from their partner while they topped-up with a big sum from their natal family. In the area, men would not easily accept financial support from the natal family as such support may be perceived as the male partner being not “man enough” to take care of his family.

*My father told me after I experienced a lot of challenges from the abusive partner*, *that I should get a sewing machine to aid me in mending children clothes*. *The machine could alternatively be used to mend other people’s clothes so that I get money and support myself as form of employment*. *I asked my husband and he agreed that we will use for mending clothes of children*, *however*, *it is keeping me busy to mend clothes of other people*. [A 31-year-old woman with primary level education] (IDI 09)

#### Mediation

Making sure that disputes are settled in the event of the relationship being near separation was reported by three participants as being the responsibility of the natal family. Going through a process of formalizing separation, if that was the course of action decided upon, was also another responsibility of the natal family. In the area, the processes relating to the union as well as separation of partners need to be negotiated among elders. One participant who was forced to leave the house after a fight and stayed with her natal relatives reported that her parents were very instrumental in negotiating her return. This was accompanied by warning the partner regarding his abusive behavior. A 26-year-old woman with primary education narrated what happened at the natal family where she was living following separation from her partner a week earlier:

*We received information from his family that he [the partner] needs to reconcile with me so that I go back to stay with him and take care of the children*. *My parents asked them to come with him for a meeting so that they hear the actual story from me and him*. *During the meeting*, *he requested forgiveness in front of his parents and my parents*. *He was told not to beat me again otherwise my parents will take me from him for good*. (IDI 01)

## Discussion

Women who are exposed to IPV during pregnancy hesitate to seek help and if they do, they are most likely to turn to their natal relatives for support. Women victims of partner violence were more likely to seek help if they considered the violence to be severe and especially when it was associated with trauma or injury. However, natal relatives and women victims of partner violence preferred to maintain their marriage. In that regard, the support provided to women victims of partner violence was geared towards making them manage the violence and continue staying in their relationship.

The findings from the present study that women victims of partner violence would seek help when the violence is considered severe is important and can be linked to the fact that violence occurred during pregnancy. Seeking help in this context may be linked with the need to seek security for themselves and their pregnancy, aiming at averting the physical consequences of violence that will happen to them and their unborn child. Studies have documented similar findings of help-seeking to occur when the victims experienced violence that they considered severe, they could no more endure, was associated with injuries and/or carried a possibility of death [33–35]. These studies have indicated that victims of partner violence were likely to tolerate violence that was less severe and non life-threatening to avoid negative consequences of help-seeking, including social stigma and partner retaliation, which have been reported to occur to other women who tried to seek help.

Consistent with other studies done in Tanzania and Nigeria, the present study has shown that some gender norms continue to make it difficult for women to seek help when they experience violence [26,36]. Cultural expectations on issues of formalizing relationships played a key role in negatively influencing help-seeking from the natal relatives among victims of violence in Moshi, Tanzania. Women who were either pregnant or decided to live with their partner before receiving formal approval by natal relatives, felt inferior and therefore hesitated to seek help. The fate of those who lived with their partners before the first dowry installment was fully paid was similar. This finding is supported by a study conducted in Tanzania, which showed that women who are considered inferior by other community members were more likely to feel ashamed to seek help and, as a result, they hide and continue suffering in silence [36]. Socially isolated women are also less likely to join supportive groups hence decreasing their chances to be supported by other informal and formal support networks [22].

It is evident from the findings of the present study that both the women victims of partner violence and the natal relatives preferred that the women stay in their relationship regardless of the severity of violence. Natal relatives did encourage women to maintain their marriage. In line with their advice, the support provided to women victims of partner violence was geared toward making them manage the violence and continue staying in their relationship. The advice from natal relatives was clear and their practical and emotional support aimed at building the victim’s resilience towards violence. Even when separation was inevitable, reconciliation or mediation was aimed at making sure that the relationship survived.

A set of reasons may explain as to why both the natal relatives and women victims of partner violence preferred to maintain the marriage, if at all possible. First is the preference for joint care of the pregnancy and the children. Although children in the study area belong to the male partner in line with the patrilineal kinship structure, the responsibility of their care is in most cases left to the female partner [37,38]. Partners’ living together is considered to be the central component of the milieu for the women to exercise their responsibility of child care in northern Tanzania. In case of separation, children are left with their father, and the mother’s responsibility of caring for her children is nearly terminated. Along similar lines, a study in Turkey documented that children were considered the property of the male partner and his family [39] and would remain in their custody in case of separation. It was further found that the natal relatives would most likely conform to the culture and send the children back to their father in case of separation. Since the women’s opportunity to care for their children is mostly provided when they are in a marriage setting, this affected women’s own view of marriage. Hollos et al., who studied issues of motherhood in Moshi, found that women considered marriage to be primarily for having children and thus, regardless of the state of affairs in the union, marriage had to continue because of children [40]. Consistent documentation is available elsewhere [33,34,41] that indicates childcare to be central to the concept of marriage and primarily the responsibility of women within the relationship. To most women victims of partner violence, separation would imply mothers running away from their core responsibility of childcare and ultimately losing their children. However, there is a great concern among stakeholders fighting violence about the well-being and future behavior of children who continue to live in such families where violence occurs, and they witness partner violence.

Circumstances may arise where the mother is granted the right to take care of children in case of separation; however, women fear to be “bad mothers” if they fail to provide good care to their children as single mothers. At the time of separation and if the age of the child is below seven years or the partner is not interested in taking care of the children, the woman may leave with her children. Supporting children’s expenses for food, clothing, school fees and medical treatment may be difficult for single women, and especially when they hold no reliable source of income. In Tanzania, children embody moral and future economic values such that every mother would like to provide the best for their children [40]. Mothers are not called by their names but rather, addressed as “the mother of the child”, indicative of how having a well-supported child earns the mother respect and confers a proper adult identity. Well-supported children are expected to take care of their aging parents, help educate their younger siblings and support community festivals. Many examples exist of street children in the area that speak for some cases where families are dissolved as a result of partner violence and the single mothers ultimately failed to take care of their children [42]. No woman would like their children to be in that situation. Other studies elsewhere have found that pregnancy earns similar respect and women would rather continue to stay in a violent relationship for the sake of caring for both the pregnancy and the child [43]. Women would therefore prioritize the mothering role–protecting and supporting their children–above all decisions within the abusive relationship [41].

The third reason that may explain why women would rather stay in an abusive relationship is the stigma around separation. In northern Tanzania where this study was conducted, separation carries negative implications about and for the natal relatives and the woman. If the woman opts for separation, the natal relatives may be required to pay back the bride wealth to the family of the partner, in part or full [44]. In most circumstances, the bride wealth may have been used by the woman’s brothers to marry or shared among the natal relatives and such a gesture morally obliges the family to look after her marriage. Separation may socially mean that the woman was not groomed well by the parents and that the family did not take care of her marriage as they are obliged to do [39]. Another study on motherhood and union formation in Moshi documented that being a mother significantly reduced the likelihood of engaging in another marital union after separation [45], denoting how separation is surrounded by stigma. Even when she is married to a new partner, the new partner will not take care of the children of a “stranger” (that is, the former male partner). Therefore, women may opt to continue staying with an abusive partner in the hope that he will later change and love her again.

It is important to point out that although most natal relatives encouraged the woman to stay in her marriage, there were also cases in which natal relatives encouraged the woman to seek a divorce. The advice of not tolerating the abusive relationship was offered by younger sisters of the abused women or their parents who were either educated or with financial capacity to support them. Younger generations and educated parents or those who are capable financially try to question gender norms that accept violence and provide to women victims of violence with alternative advice to leave the abusive relationships. However, the final decision of leaving the abusive relationship rests with the abused—who are generally entrapped within the cultural expectations of joint child care which stigmatize separation and foster maintenance of marriage regardless of violence.

There is a need to point to the strengths and limitations of the present study. This qualitative study was nested within a bigger cohort study, and as a strength, allowed the use of validated tools in assessing violence. Further, the qualitative approach allowed us to build rapport with the study participants, enabling them to tell their stories in their own words. However, it remains true that gender based violence is a sensitive topic to explore in Tanzania. Frequent contacts with the participants during the cohort study and during qualitative interviews created opportunities for building trust between participants and interviewers while facilitating free expression. Another limitation of the study is the qualitative nature of the research, which limits the generalization of the findings to all areas in Tanzania. Instead, efforts have been made to discuss the results of the present study by comparing and contrasting with other studies done elsewhere, noting that the results are in line with what has been documented in other settings characterized by similar cultural and gender norms. Due to the limited number of participants who reported other sources of support, it was also not possible to provide in-depth information on other forms of social support, such as support provided by friends, in-laws, or community members. These forms of support are important topics for future research in Tanzania and other settings.

## Conclusion

This study has found that many women who are exposed to IPV during pregnancy hesitate to seek help and, if they do, they are most likely to turn to their natal relatives for support. The natal relatives are willing to provide support, though they strongly encourage women to maintain their marriage so that they continue caring for their children jointly with their partners. Emotional support was the most common form of support provided and included showing love, empathy and praying. Information was another form of support and it aimed mainly at encouraging the women to maintain their marriage so as to care for their children. Practical support included direct financial support and building their economic base to reduce dependency on their partners. When a couple was on the verge of separation, reconciliation or mediation was provided by the natal family to save the marriage. This indicates that in this cultural setting of northern Tanzania, people tend to give children and family the highest priority. As a consequence, it is likely that many women will continue to live in violent domestic situations. Stakeholders supporting victims of violence in Tanzania and similar areas need to understand the fears and priorities of victims of violence and structure interventions to address these priorities, for instance by offering legal assistance in child custody cases; helping women to become financially independent; conducting communication campaigns to address the stigma that surrounds separation; or, legal assistance for women who prefer to stay in their marriages, offer counseling on how to handle marital difficulties. Given the key role of natal relatives in providing support to women who live with IPV, it is important that not only the women themselves, but also their natal relatives are actively involved by stakeholders in interventions that aim to assist women who experience partner violence.
